# Pneumothorax in a Preterm Neonate: A Case Report

**DOI:** 10.31729/jnma.5819

**Published:** 2021-06-30

**Authors:** Rajan Phuyal, Ritika Basnet, Abhin Sapkota, Uttara Gautam, Vijaya Kumar Chikanbanjar

**Affiliations:** 1 Department of Pediatrics, Kathmandu Medical College Teaching Hospital, Sinamangal, Kathmandu, Nepal; 2 Manakamana Hospital Private Limited, Jhapa, Nepal

**Keywords:** *needle thoracostomy*, *pneumothorax*, *preterm infants*

## Abstract

A pneumothorax is an abnormal collection of air in the pleural space between the lung and chest wall. Although this condition commonly occurs in adults, it can also present as complication in neonates requiring assisted ventilation and has high morbidity and mortality. Chest tube placement and needle drainage are some common approaches in management. A late preterm infant born at 35+2 weeks of gestation was admitted in Neonatal Intensive Care Unit for the management of respiratory distress. He was kept on mechanical Continuous Positive Airway Pressure owing to worsening respiratory distress. Chest X-ray revealed pneumothorax that was successfully managed with venous catheter drainage on second intercostal space with underwater seal. He was discharge on 10th day of Neonatal Intensive Care Unit admission with stable vitals and normal breathing pattern.

## INTRODUCTION

Pneumothorax is a condition where there is free air in the pleural space. In preterm neonate, it has been associated with an increased risk of intraventricular hemorrhage and mortality.^1,2^ Although the use of postnatal surfactant has reduced the risk of pneumothorax in neonates,^3,4^ it remains an important complication. Timely diagnosis and appropriate treatment are crucial to reduce complications and mortality due to pneumothorax. Here, we present a case of management of pneumothorax with a venous catheter inserted in second space with underwater seal in a late preterm with respiratory distress that was kept on mechanical continuous positive airway pressure.

## CASE REPORT

A preterm male neonate was delivered to multipara mother at 35 weeks and two days of gestation via emergency lower segment caesarean section for fetal distress. Baby had an APGAR score of 8/10 and 9/10 at 1 minute and 5 minutes respectively and birth weight of 2.2kg. Baby was admitted in NICU for further management. Baby developed tachypnea up to 80 breaths per minute for which baby was kept on Mechanical Continuous Positive Airway Pressure (CPAP) at Positive End Expiratory Pressure (PEEP) of 5 cm of H_2_O. Surfactant was not considered as the baby was maintaining oxygen saturation at FiO_2_ of 25% and chest X-ray was normal. IV antibiotics Ampicillin and Amikacin were started as per unit protocol in view of raised C-reactive protein. Other parameters of initial sepsis screening were normal.

**Figure 1:**
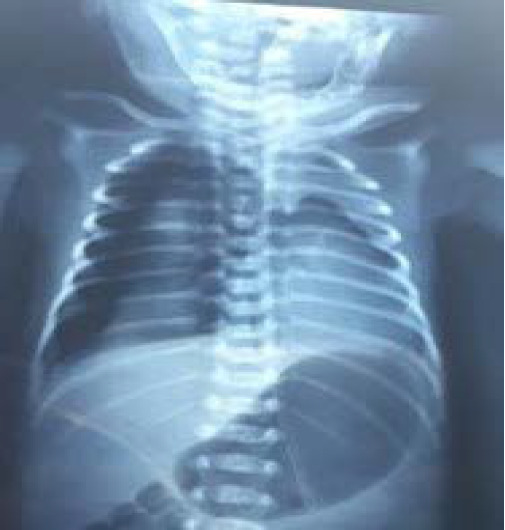
Right sided pneumothorax with mediastinal shift.

At 19^th^ hour of life, baby had worsening respiratory distress with increased tachypnea, severe intercostal, sub-costal and supra-sternal retractions. The FiO_2_ requirement increased up to 50%. An urgent chest X-ray was done which revealed a right-sided pneumothorax along with mediastinal and tracheal shift to the opposite side which was suggestive of tension pneumothorax (Figure 1).

**Figure 2:**
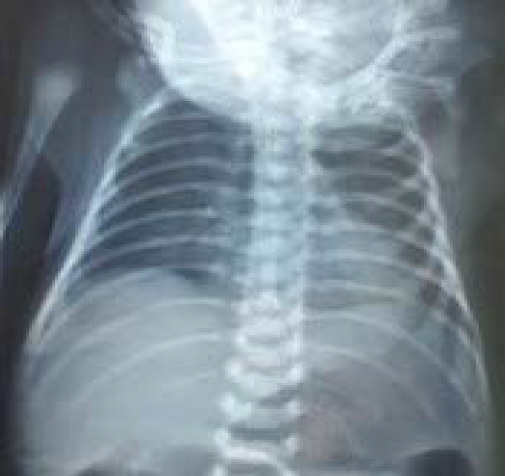
Repeat chest X-Ray showing resolved pneumothorax.

As tension pneumothorax is an emergency condition it was managed with IV cannula drainage at second intercostal space and which was later connected to an underwater seal. Respiratory distress began to subside significantly. Antibiotics was upgraded to Piperacillin/Tazobactam and baby was kept nil per oral. The IV cannula placed in the chest was removed accidently at 42^nd^ hour of life. There was no worsening of tachypnea, chest retraction and laboured breathing. FiO_2_ requirement and Silverman score significantly decreased. Chest X-ray was repeated after four hours which showed resolving pneumothorax (Figure 2).

Feeding was gradually increased without complications and kept on full feed. As the respiratory distress settled and FiO_2_ requirement decreased, baby was weaned to nasal prong on 5^th^ day of life, baby was comfortable on room air on 7^th^ day of life. Baby was successfully discharged on 10^th^ day with stable vitals, normal respiratory pattern and activity.

## DISCUSSION

Pneumothorax in preterm neonates with respiratory distress vary from 3% (receiving mechanical ventilation) to 9% (receiving CPAP support).^6^ Worsening tachypnea, chest retractions, nasal flaring, grunting and cyanosis are few clinical signs of pneumothorax in a neonate. Respiratory distress syndrome, meconium aspiration syndrome, pneumonia and transient tachypnea of newborn are some of the risk factors. Mortality is very high when pneumothorax is untreated or treatment is delayed. The development of a pneumothorax with ensuing hypoxia and hypercapnia is a potentially life-threatening condition and 30% of the infants die in NICU.^7^ In our case, early recognition and treatment of pneumothorax was key in good outcome. Pneumothorax is most commonly encountered in the first three days. This is probably due to high transpulmonary pressure caused by the onset of new breathing.^8^ Our case was identified as pneumothorax at 19^th^ hour of life.

Chest tube placement is the most common treatment approach in management of severe neonatal pneumothorax. It involves surgical procedure and various complications. Few trials support insertion of a venous catheter as a safe alternative to chest tube placement as a method of draining air from neonates with pneumothorax. This is an easy and quick bedside procedure and is particularly useful for neonates that require immediate air drainage.^9^ As compared with chest tube drainage, needle aspiration decreased hospital stay and decreases surgery related complication. Our case was also managed with insertion of 22-gauge cannula connected to an underwater seal. There was significant improvement in respiratory rate, chest retraction, Silverman score and Fi02 requirement after needle insertion. It also avoided chest tube insertion and patient was discharged on 10^th^ day of hospital admission.

Another alternative to chest tube placement in neonate is high-frequency oscillatory ventilation that provides a conservative management in a case of significant pneumothorax in a preterm neonate who is hemodynamically stable and requires mechanical ventilation.^10^

Pneumothorax in a neonate is a serious condition with high morbidity and mortality which requires early identification and prompt management. Venous catheter with underwater water seal can be considered as an alternative to chest tube drainage to minimize surgical complication and hospital stay.

## References

[1] Hill A, Perlman JM, Volpe JJ (1982). Relationship of pneumothorax to occurrence of intraventricular hemorrhage in the premature newborn. Pediatrics.

[2] Powers WF, Clemens JD (1993). Prognostic implications of age at detection of air leak in very low birth weight infants requiring ventilatory support. J Pediatr.

[3] Malek A, Afzali N, Meshkat M, Yazdi NH (2011). Pneumothorax after mechanical ventilation in newborns. Iran J Pediatr.

[4] Morley CJ (1997). Systematic review of prophylactic vs rescue surfactant. Arch Dis Child Fetal Neonatal Ed.

[5] Horbar JD, Badger GJ, Carpenter JH, Fanaroff AA, Kilpatrick S, LaCorte M (2002). Trends in mortality and morbidity for very low birth weight infants, 1991-1999. Pediatrics.

[6] Morley CJ, Davis PG, Doyle LW, Brion LP, Hascoet JM, Carlin JB (2008). Nasal CPAP or intubation at birth for very preterm infants. N Engl J Med.

[7] Ozer EA, Ergin AY, Sutcuoglu S, Ozturk C, Yurtseven A (2013). Is pneumothorax size on chest x-ray a predictor of neonatal mortality?. Iran J Pediatr.

[8] Apiliogullari B, Sunam GS, Ceran S, Koc H (2011). Evaluation of neonatal pneumothorax. J Int Med Res.

[9] Arda IS, Gurakan B, Alíefendíoglu D, Tuzun M (2002). Treatment of pneumothorax in newborns: use of venous catheter versus chest tube. Pediatr Int.

[10] Aurilia C, Ricci C, Tana M, Tirone C, Lio A, Gambacorta A (2017). Management of pneumothorax in hemodynamically stable preterm infants using high frequency oscillatory ventilation: report of five cases. Ital J Pediatr.

